# Sensitive Materials Used in Surface Acoustic Wave Gas Sensors for Detecting Sulfur-Containing Compounds

**DOI:** 10.3390/polym16040457

**Published:** 2024-02-06

**Authors:** Yuhang Wang, Cancan Yan, Chenlong Liang, Ying Liu, Haoyang Li, Caihong Zhang, Xine Duan, Yong Pan

**Affiliations:** 1 School of Chemistry and Chemical Engineering, Shanxi University, Taiyuan 030006, China; 202222902021@email.sxu.edu.cn (Y.W.); ly19861900620@163.com (Y.L.); vc766693@163.com (H.L.); duanxe@sxu.edu.cn (X.D.); 2 State Key Laboratory of NBC Protection for Civilian, Beijing 102205, China; ccy805905145@163.com; 3 Institute of Acoustics, Chinese Academy of Sciences, Beijing 100190, China; liangchenlong22@mails.ucas.ac.cn; 4 School of Electronic, Electrical and Communication Engineering, University of Chinese Academy of Sciences, Beijing 100049, China

**Keywords:** polymer, films, surface acoustic wave (SAW), gas sensor, mustard gas, hydrogen sulfide, sulfur dioxide

## Abstract

There have been many studies on surface acoustic wave (SAW) sensors for detecting sulfur-containing toxic or harmful gases. This paper aims to give an overview of the current state of polymer films used in SAW sensors for detecting deleterious gases. By covering most of the important polymer materials, the structures and types of polymers are summarized, and a variety of devices with different frequencies, such as delay lines and array sensors for detecting mustard gas, hydrogen sulfide, and sulfur dioxide, are introduced. The preparation method of polymer films, the sensitivity of the SAW gas sensor, the limit of detection, the influence of temperature and humidity, and the anti-interference ability are discussed in detail. The advantages and disadvantages of the films are analyzed, and the potential application of polymer films in the future is also forecasted.

## 1. Introduction

### 1.1. The Fundamental Concepts of SAW Sensors

In 1885, for the first time, British physicist Rayleigh discovered the surface acoustic wave (SAW), which was a type of elastic mechanical wave propagating along the surface of an elastic object, while he studied seismic waves [1]. Due to technological limitations at that time, SAWs were not used in practical applications. In 1965, the invention of interdigital transducers (IDTs) by White R.M. and Voltmer F.W. provided a simpler method of generating SAWs and accelerated the development of the SAW sensor [2]. Since the publication of the first paper on SAW gas sensors by Wohltjen H. and Dessy R. in 1978, SAW gas sensors have been extensively studied [3,4,5]; over the past 40 years, they have been used to detect many kinds of hazardous gases such as SO_2_, H_2_S, NO_2_, NH_3_, methane, hydrogen, explosives, and chemical warfare agents [6]. Because of advantages of a small size, high sensitivity, ease of integration, intelligence, and low-cost mass production, more and more researchers all over the world have paid much attention to this new field.

A SAW sensor is mainly composed of IDTs and piezoelectric materials (e.g., quartz, LiNbO_3_, LiTaO_3_, ZnO, AlN, Bi_12_GeO_20_, AsGa, piezoceramics, etc. [7,8,9,10]). When a sinusoidal wave with the same period as the IDT is applied to the input IDT, vibration will generate under the IDT, resulting in the generation of a SAW perpendicular to the IDT; the SAW propagates along the piezoelectric material in the direction away from the input IDT; when the SAW reaches the output IDT, the output IDT converts the acoustic waves to electrical signals via the piezoelectric effect [11]. The energy of SAW is primarily limited to the surface of elastic objects, and tiny variations in the surface, such as changes in temperature, pressure, and weight, will alter the acoustic wave signals received by the output IDT [12], which result in the high sensitivity of SAW gas sensors.

According to distinct structures and operating principles, SAW sensors can be categorized as a delay-line-type and resonator-type [13]. For dual-delay lines, one is coated to be used to detect harmful gases, and the other is used as a reference channel to reduce the influence of environmental factors, such as temperature or pressure. Resonator-type sensors have a high Q value, low insertion loss, and small frequency drift, which can further enhance their sensitivity and distinguishability despite their more complicated mechanism and structure [14]. In terms of functionality, SAW sensors are classified into three categories: physical sensors, chemical sensors, and biosensors. Physical sensors are primarily used to detect physical parameters such as temperature and pressure, and chemical sensors are often used to detect gases qualitatively and quantitatively, while biosensors are employed to detect substances such as deoxyribonucleic acid (DNA), proteins, etc. [15].

**Figure 1:**
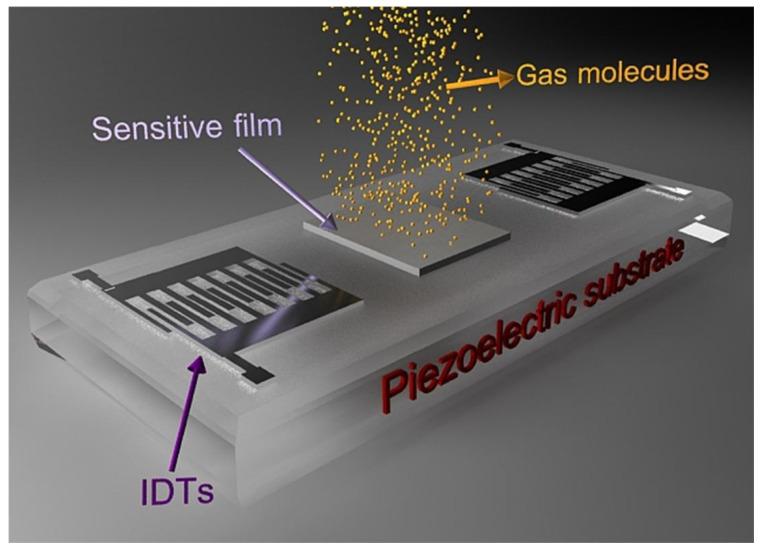
Scheme of SAW delay line [18]. Reproduced with permission from Mohsen Asad, Surface acoustic wave based H_2_S gas sensors incorporating sensitive layers of single wall carbon nanotubes decorated with Cu nanoparticles; published by Sensors and Actuators B: Chemical, 2014.

For a SAW chemical gas sensor, such as the delay line shown in Figure 1, the SAW might be generated from the input IDT and pass through the surface of the sensitive film covering on the piezoelectric material; when the SAW reaches the output IDT and adsorbs a certain amount of gas, a frequency shift occurs. In general, the sensitive material can adsorb target gases selectively and reversibly in two ways, physical adsorption or chemical adsorption, that is, van der Waals force, hydrogen bonds, or a chemical reaction between the target gas and film, and sometimes, the solubility of the target gas in the film is considered [11], which alters the film’s physicochemical parameters, such as the mass, density, modulus of elasticity, or conductivity, and affects the wave velocity or frequency of the passing SAW [16,17]. In the detection of toxic or harmful gases with SAW sensors, the physical and chemical properties of the sensitive materials and the selection of coating conditions (e.g., film thickness, surface roughness, etc.) will have great influence on the sensitivity, selectivity, repeatability, and stability of the sensors; therefore, optimizing membrane materials is very important for preparing a SAW sensor.

### 1.2. Sulfur-Containing Hazardous Gas Species

#### 1.2.1. Sulfur-Containing Chemical Agents

Mustard gas (HD), a vesicant chemical agent, is regarded as the king of toxic agents due to its ability to induce necrosis and tissue degeneration. As a lipophilic vesicant, it can permeate the body via the skin, eyes, and breathing system. Its alkylation reaction with proteins, DNA, and glutathione will cause cellular damage [19]. Although mustard gas has a lethality rate of only 2–5%, it has a high morbidity and psychological impact on people; this is because it has a median lethal dose (LD_50_) of about 100 mg/kg, a median lethal concentration (LCt_50_) of 15,000 mg min/m^3^ [20], and a minimum dose of 0.2 mg to cause skin blistering [21], and the most important thing is that there is no effective antidote or treatment. Mustard gas was first synthesized by Despretz in 1822 and used in war in 1917 [22]; it caused about 1.3 million injuries and over 90,000 deaths during the First World War [23], mustard gas was also used in the Second World War and the Iran–Iraq War. According to the Chemical Weapons Convention which came into force in 1997, chemical weapons should be eliminated within ten years; however, because of the legacy of war and the fact that chemical weapons are cheap and easy to produce [24], research on detecting chemical agents are still ongoing. Table 1 lists common chemical agents along with their simulants.

#### 1.2.2. Sulfur-Containing Harmful Gas

Hydrogen sulfide (H_2_S), an acidic, toxic, and flammable gas, has an LD_50_ of 673 mg/kg and an odor threshold for H_2_S of 11 ppb, but olfactory paralysis happens at a concentration of H_2_S higher than 140 ppm [28]; people will collapse within 5 min, suffer serious eye impairment within 30 min, and face the risk of death after 30 to 60 min when the concentrations of H_2_S reach 500–700 ppm [29]. H_2_S naturally exists in volcanic eruptions, paper making, coal mining, chemical production, automobile exhausts, etc. Since it is one of the main causes of environmental pollution, it is essential to monitor the concentration of hydrogen sulfide gas in real time. Sulfur dioxide (SO_2_), a colorless gas with an irritating odor, is one of the major pollutants in the atmosphere, it is mainly produced by natural or artificial processes such as burning fossil fuels containing sulfur (for example, coal, oil, and natural gas), volcanic eruptions, and smelting and forging sulfur-containing minerals [30]. Prolonged exposure to SO_2_ can cause harm to the eyes, lungs, and throat. Additionally, SO_2_ easily dissolves in water to form acid rain, which severely threatens buildings, plants, animals, and overall environmental balance [31]; therefore, monitoring SO_2_ is an essential aspect of environmental protection.

## 2. Sensitive Functional Materials of Sulfur-Containing Agents and Their Simulants

### 2.1. Polymer

In 1993, Grate et al. [32] employed a 158 MHz four-channel SAW delay line sensor array to detect mustard gas and sarin. Poly(epichlorohydrin) (PECH), poly(ethylenimine) (PEI), ethyl cellulose (ECEL), and fluoropolyol (FPOL) were utilized as the sensitive films of the sensor array, and signal processing and pattern-recognition algorithms were also employed to discriminate the target gases. Without preconcentration, mustard and sarin could be detected at concentrations as low as 2 mg/m^3^ and 0.5 mg/m^3^, respectively; however, when the 2 min preconcentration mode was used, the detection limits could be improved to 0.5 mg/m^3^ for mustard gas and 0.01 mg/m^3^ for sarin. This proved that the preconcentration mode enhanced the sensitivity of the sensors. Additionally, the study also discovered that the channel coated with PECH was more sensitive to mustard gas when humidity levels increased; however, the specific mechanism through which humidity influenced the sensor was not clearly explained and still needed to be further investigated.

In 2005, Liu et al. [33] used a 159 MHz SAW dual delay line coated with PECH as a sensitive film to detect mustard gas. To enhance the performance of sensors, the correlation between film thickness and sensitivity was investigated, the findings revealed that sensitivity increased with an increase in the film thickness. In the same year, Liu et al. [34] conducted a response test on the same sensor and found a good linear relationship between HD concentrations in the range of 2–200 mg/m^3^ and the corresponding signals. The sensitivity of the sensor was determined to be 170.1 Hz/(mg/m^3^). Additionally, they also found that when the temperature increased from 0 °C to 50 °C at a concentration of 2 g/m^3^ of CEES, the frequency shifts decreased by 95%, and the response time and the recovery time become shorter when temperature increased. Additionally, a repeatability test showed excellent performance of the prepared sensor. In 2006, Liu et al. [35] investigated the adsorption kinetics between a PECH film and mustard gas with multimolecular layer adsorption model, they concluded that gas/liquid balance theory and van der Waals forces was very important for physical adsorption, and the related work in [36] was also summarized.

In 2007, Chen et al. [37] used an array that consisted of four 200 MHz two-port resonators with four different polymers (PECH, Silicone (SE-30), Hexafluoro-2-propanol bisphenol-substituted siloxane polymer (BSP3), fluorinated polymethyldrosiloxane (PTFP)) to detect HD, DMMP, GB, and sarin acid. Combined with a probabilistic neural network (PNN), the recognition rate could reach 90.87% successfully, and mustard gas was well recognized. In 2008, the stability, sensitivity, repeatability, consistency, and selectivity of a SAW PECH sensor were evaluated by this team [14]; the repeatability and consistency were found to have relative standard deviations of 3.27% and 2.50%, respectively, which were within the margin of error, and the detection limit was 0.3 mg/m^3^. 

**Figure 2:**
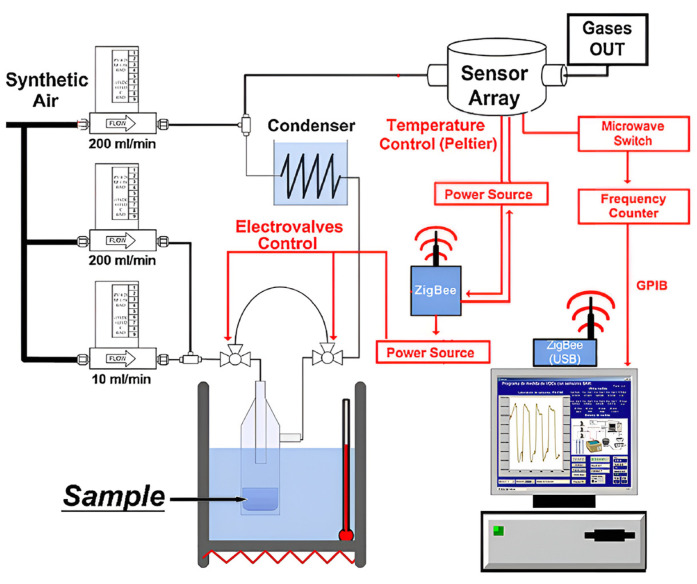
Scheme of the instrumentation used for data acquisition in real time [39]. Reproduced with permission from Matatagui D., Array of Love-wave sensors based on quartz/novolac to detect CWA simulants; published by Talanta, 2011.

In 2009, Matatagui et al. [38] employed a 157 MHz six-channel SAW delay line sensor array with an electrode thickness of 200 nm and a finger spacing of 5 μm to detect DMMP, DPGME, DMA, and DCE. Six polymers, including PECH, polycyanopropylmethylsiloxane (PCPMS), carbowax, polydimethylsiloxane (PDMS), PEI, and trifluoropropylmethylsiloxane–dimethylsiloxane (PMFTPMS), were prepared on the delay line, and the sensor array exhibited a rapid and significant response. The data obtained from the array were analyzed using principal component analysis (PCA) and PNN, which resulted in excellent distinguishability and a low detection limit. In their 2011 study, Matatagui et al. [25] successfully detected several substances with the same devices, including DMMP, DPGME, toluene, DCM, DCP, DMA, and DCE, they concluded that all simulants were accurately identified except DCE and DCM, as these two substances had very similar structures and could not be distinguished. In the same year, Matatagui et al. [39] developed a six-channel delay line array based on the Love wave; the sensor array was prepared with an aluminum electrode with a thickness of 200 nm and a finger spacing of 7 μm. Through spin-coating, a Novolac photoresist guide layer with a thickness of 0.8 μm was applied onto the surface of the piezoelectric material. Subsequently, the sensitive materials mentioned above were prepared on the surface of the delay line. DMMP, DPGME, DMA, DCE, DCM, and DCP were tested with the detection system, and their gas concentration and temperature were controlled, as shown in Figure 2. The sensor array exhibited excellent stability, reversibility, repeatability, and sensitivity. The CWA simulants were also accurately detected and categorized with PCA and PNN. In 2012, the same team [40] utilized a 3-micron-thick SiO_2_ guide layer acquired through plasma-enhanced chemical vapor deposition to detect the same six target gases, and the results showed that the detection limits were 0.04 ppm, 0.25 ppm, 15 ppm, 75 ppm, 125 ppm, and 5 ppm, respectively.

In 2011, He et al. [41] designed a novel 300 MHz SAW dual delay line. The device was prepared with an Al/Au electrode structure and strategic phase modulation to minimize insertion loss. A PECH film was applied by solvent evaporation, and the thickness of the film was about 80 nm. This was performed under the conditions of 24 °C, RH 50%, sensor sensitivity of 25 Hz/(mg/m^3^) to mustard gas, a linear range of 2–200 mg/m^3^, and a repeatability error of ±10%. In 2017, Qi et al. [42] designed a 3D nanocluster resonator sensor whose surface was modified by ZnO nanoclusters to provide a larger specific surface area for the sensitive layer, thus increasing the detection sensitivity; however, it also led to an increase in the insertion loss of the sensor. When PECH, SE-30, PTFP, and BSP3 were used as sensitive films to detect a mixture of mustard gas and sarin, it obtained an identification rate of over 90%.

In 2018, Pan et al. [43] developed a SAW sensor array with a wireless communication network module and a positioning system module; in this sensor array, PECH, triethanolamine, fluoroalcoholpolysiloxane, and L-glutamic acid hydrochloride were used as sensitive films to detect CEES, H_2_S, DMMP, and NH_3_, respectively. Combined with pattern-recognition algorithms, target gases were successfully detected at safe concentrations outside within a range of 300 m. This study demonstrated the feasibility of using wireless sensor networks for gas detection. In light of the absence of prior research on the influence of temperature and humidity, Pan et al. [6] conducted a study in 2020 to explore the environmental adaptability of the PECH-SAW sensor in detecting CEES. The findings revealed that as the ambient temperature rose, the sensor’s response value decreased, and the response time shortened. On the other hand, the detection signal exhibited an apparent increase in a higher-humidity environment; this phenomenon was attributed to the elevated environmental humidity, which amplified the solvation impact of CEES on PECH and facilitated the creation of hydrogen bond active sites. The sensor showed excellent selectivity and resistance to interfering gases in a smoke test, and its sensitivity of 233.17 Hz/(mg/m^3^) along with stability over 18 months were also investigated. In 2022, Pan et al. [44] studied the physical characteristics of PECH film in detail. A viscosity of 1.969 was obtained and the glass transition temperature was found to be as low as −22.4 °C. At same time, the work of adhesion, work of immersion, and spreading coefficient were calculated, too. In general, the linear solution–energy relationship (LSER) (Equation (1)) is often used to evaluate the adsorption ability between films and target gases, and the related LSER parameters of PECH are summarized in Table 2.

(1) $$\log\;K=c+rR_{2}+s\pi_{2}^{H}+a\sum \alpha_{2}^{H}+b\sum \beta_{2}^{H}+l\;\log\;L^{16}$$

**Figure 3:**
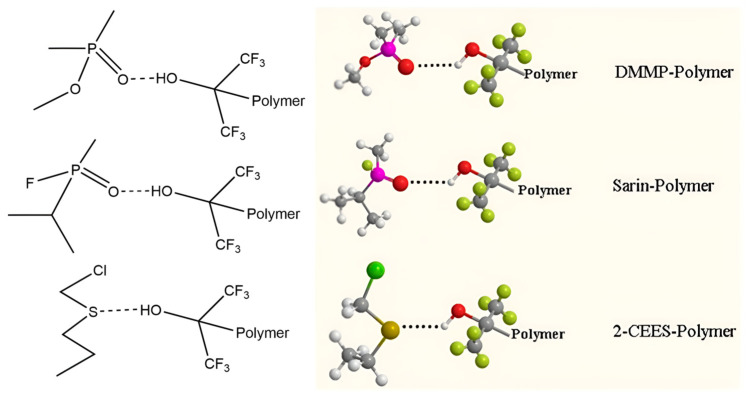
Hydrogen-bonding interactions between DMMP, sarin, 2-CEES, and HBA polymer PLF [47]. Reproduced with permission from Yin Long, The different sensitive behaviors of a hydrogen-bond acidic polymer-coated SAW sensor for chemical warfare agents and their simulants; published by Sensors, 2015.

There have also been some reports on using other polymers to detect mustard gas and its simulants. In 2000, McGill et al. [45] employed an alarm system called SAWRHINO which utilized three unspecified polymer materials to detect HD, DMMP, and GD, and this is one of the few devices to use SAW technology in practice so far. In 2006, Shi et al. [11] developed liquid-phase macromolecular synthesis technology to implement molecular-level doping of poiyaniline (PANI) and phthalocyanine palladium (PdPc), which resulted in the creation of a novel organic semiconductor-sensitive material called PdPc_0.3_PANI_0.7_. It was observed that the material exhibited stability at a temperature of 300 °C by employing differential thermal analysis. The PdPc_0.3_PANI_0.7_ compound was applied onto the surface of a SAW dual delay line using vacuum-coating technology. The sensor exhibited high sensitivity of 105 kHz/(mg/m^3^), and the response time was less than 5 min in detecting mustard gas. In 2014, Matatagui et al. [46] fabricated a 163 MHz six-channel sensor array; the nanofibers used in this array were prepared by electrospinning technology using polyvinyl alcohol (PVA), polyvinylpyrrolidone (PVP), polystyrene (PS), PVA+SnCl_4_, PVA+SnCl_4_ annealed for 4 h at 450 °C, and the copolymer PS+Poly(styrene-alt-maleic anhydride) (PS+PSMA) for detecting DMMP, DPGME, DMA, and DCE. The linear relationship between the concentration and response was found, and it was also proven that it was possible to achieve an identification rate of 100% by employing PCA. In 2015, Long et al. [47] applied a strong hydrogen-bond acidic (HBA) polymer linear fluoroalcoholic polysiloxane (PLF) as the sensitive material to detect GB, DMMP, HD, CEES, and DCP, as depicted in Figure 3. The sensor exhibited a significant response to sarin, DMMP, and CEES, while a minimal response to mustard gas and DCP was also found. The difference that existed in mustard gas, DCP, and CEES might be due to the differing polarity and electron cloud distribution of mustard gas, DCP, and CEES; this was believed to result from the chlorine atoms’ strong electronegativity and the sulfur atoms’ electron richness. The factors discussed above affected the formation of hydrogen bonds and diminished the detection effectiveness, so the sensor was deemed unsuitable for detecting mustard gas.

### 2.2. Organic Small Molecule

**Figure 4:**
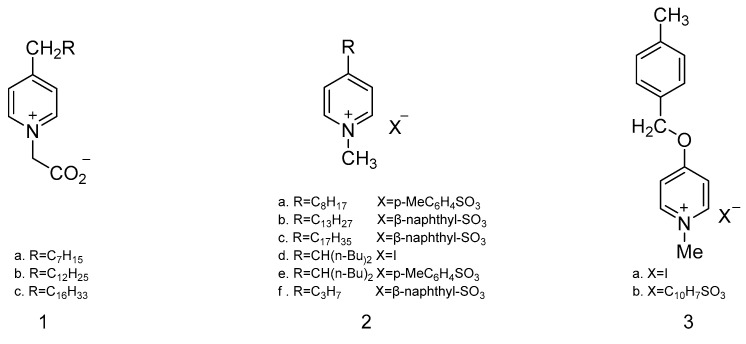
The structures of pyridinium betaine (**1**), pyridinium salt (**2**), and pyridine ether (**3**) [48]. Reproduced with permission from Katritzky A.R., Utilization of pyridinium salts as microsensor coatings; published by Langmuir, 1989.

**Figure 5:**
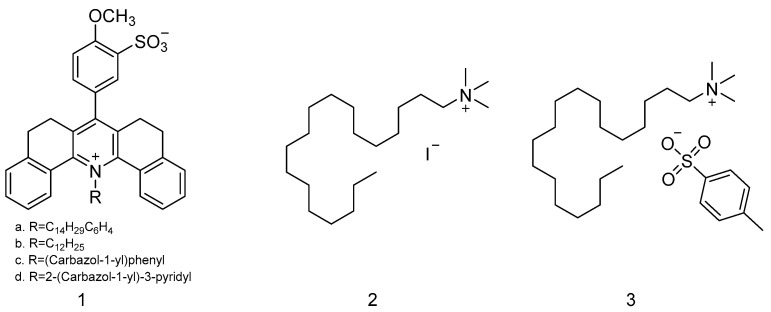
The structures of acridinium betaine (**1**) and two quaternary ammonium salts (**2**,**3**) [49]. Reproduced with permission from Katritzky A.R., Synthesis and response of new microsensor coatings-II Acridinium betaines and anionic surfactants; published by Talanta, 1990.

Katritzky et al. prepared a SAW sensor coated with organic small-molecule sensitive films to detect mustard gas and its simulants. As seen in Figure 4, pyridine 1-oxide, pyridinium salts, pyridinium betaine compounds, pyridyl ethers, and pyridinium compounds were synthesized as sensitive materials by this team in 1989 [48]. When detecting DMMP, CEES, and H_2_O, it was found that pyridinium betaine and pyridinium sulfonate produced significant resistance changes to DMMP and CEES, respectively; however, no significant frequency shift was observed. From the point of view of the resistance response, pyridine derivatives were more easily influenced by humidity, so environmental conditions would limit their practical application. In response to this challenge, the team extended their research to acridinium betaines in 1990 [49], as shown in Figure 5, aiming to reduce humidity interference through an additional hydrocarbon mass around the ionic site. In addition to acridinium betaines, they also synthesized quaternary ammonium salts (Figure 5(**2**,**3**)) as sensitive materials to detect DMMP, CEES, and H_2_O; they found that the compounds in Figure 5(**2**,**3**) had small frequency shifts and large resistance responses to CEES, but the compound in Figure 5(**3**) reacted almost as much to water vapor as CEES. For the compounds in Figure 5(**1**a,**1**b), CEES could be detected at frequency shifts of 9.8 kHz and 6.8 kHz, respectively, but the frequency shift resulting from the film in Figure 5(**1**a) was irreversible. 

**Figure 6:**
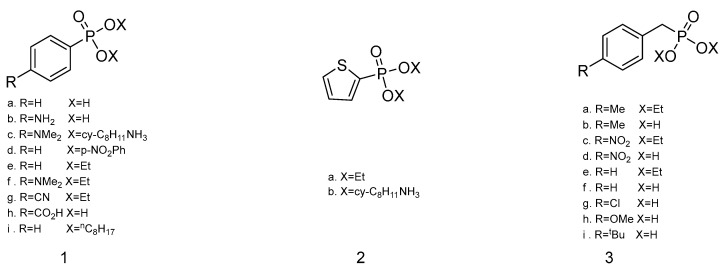
The structures of 4-substitutedphenylphosphonate derivatives (**1**), 2-thienylphosphonate derivatives (**2**), and 4-substitutedbenzylphosphonate derivatives (**3**) [26]. Reproduced with permission from Katritzky A.R., Synthesis of new microsensor coatings and their response to vapors-III arylphosphonic acids, salts and esters; published by Talanta, 1990.

**Figure 7:**
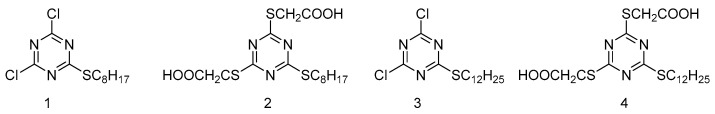
The structures of 2,4,6-trisubstituted-1,3,5-triazine derivatives (**1**–**4**) [50]. Reproduced with permission from Katritzky A.R., Synthesis of new microsensor coatings and their response to test vapors 2,4,6-trisubstituted-1,3,5-triazine derivatives; published by Talanta, 1991.

Based on the speculation of the relationship between the adsorption mass and solubility of the sensitive membrane to the measured gases, in 1990, Katritzky et al. [26] sprayed phosphonic acid, phosphonate ester, and ammonium cyclohexylphosphonate, respectively, on a SAW surface, as shown in Figure 6, for the detection of DMMP, CEES, and H_2_O. They expected that the sensor would achieve a better effect for DMMP than CEES and H_2_O. However, the results revealed that only 4-methylbenzylphosphonic acid in Figure 6(**3**b) produced a maximum response frequency of 74.3 kHz for DMMP, while diethyl 4-dimethylaminophenylphosphonate in Figure 6(**1**f) and diethyl 2-thienylphosphonate in Figure 6(**2**a) gained frequency shifts of 77.5 kHz and 65.6 kHz for CEES, respectively, and the two compounds did not exhibit a good response for DMMP and H_2_O. In 1991, they [50] employed a 52 MHz dual delay line SAW sensor and utilized several synthetic trisubstituted 1,3,5-triazines as sensitive materials to detect DMMP, CEES, and H_2_O. As shown in Figure 7, 2,4-di(carboxymethylthio)-6-octanethio-1,3,5-triazine in Figure 7(**1**) and 2,4-di(carboxymethylthio)-6-dodecanethio-1,3,5-triazine in Figure 7(**2**) showed significant frequency and resistance shifts due to the interaction of carboxylic acid and phosphate functional groups. The compounds 2,4-dichloro-6-dodecylthio-1,3,5-triazine in Figure 7(**3**) and 2,4-dichloro-6-octylthio-1,3,5-triazine in Figure 7(**4**) exhibited a 37.4-fold and 34.0-fold increase in resistance to CEES, respectively. Katritzky et al. have conducted many studies on the sensitivity mechanism of sensors and the design of functional materials, and their works have great reference value for the design of sensitive materials for the detection of toxic or harmful gases.

### 2.3. Other Kinds of Sensitive Materials

In 2013, to detect DMMP, diethyl cyanophosphonate, and the mustard gas simulants DBS and CEPS, Raj et al. [27] designed an electronic nose (E-nose) with four SAW sensors coated with ZnO, TeO_2_, SnO_2_, and TiO_2_. The four simulants of CWA were effectively distinguished with the PCA. All simulants were clearly distinguished despite including interfering substances such as petrol, diesel, kerosene, volatile organic compounds, and water vapors in the PCA. In 2016, Sayago et al. [51] attempted to develop a Love wave sensor using graphene oxide as a sensitive film. The sensor presented good reproducibility in the detection of DMMP, DPGME, DMA, and DCE. The detection limit for DMMP was 9 ppb, and the response of graphene oxide to DMMP was much greater than that of the other gases measured, which may be due to the formation of hydrogen bonds between DMMP and graphene oxide.

There have also been some reports about the detection of sulfur-containing gases by SAW devices without a sensitive film. In 2021, Fahim et al. [52] developed an uncoated resonator SAW sensor to measure the frequency changes during programmed temperature increases to detect CEES, methyl salicylate, and DMMP. The system, combined with PCA, could identify high and sub-ppm concentrations of gases, which provided a novel method for identifying compounds. In 2022, Kumar et al. [53] investigated the impact of carrier gas on detecting sensitivity by combining gas chromatography with a SAW sensor; they used H_2_, He, N_2_, and air as carrier gases for the detection of CEES, DMMP, diethyl cyanophosphonate, and triethyl phosphate, as well as methanol, toluene, and xylene. The experiments revealed that higher sensitivity could be obtained with H_2_ as the carrier gas in detecting all target gases (H_2_ > He > air > N_2_); therefore, it was judged that the sensitivity was affected by the density of the carrier gas.

## 3. Sensitive Functional Materials of Sulfur-Containing Harmful Gases

### 3.1. Sensitive Functional Materials for SO_2_ Detection

**Figure 8:**
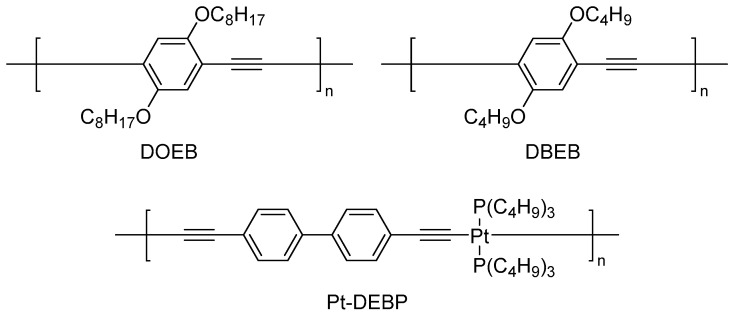
Three types of “rod-like” polymers [55]. Reproduced with permission from Penza M., SAW chemical sensing using poly-ynes and organometallic polymer films; published by Sensors and Actuators B: Chemical, 2001.

Following the development of SAW sensor technology, more research has focused on its application in detecting SO_2_. Most sensitive materials achieve an interaction with SO_2_ through the attraction between acidic gas and alkaline sites. With a tertiary amino group as the alkaline adsorption center, N,N-dimethyl-3-aminopropyltrimethoxysilane (NND) has been a well-known material for detecting SO_2_. In 1996, Leidl et al. [54] combined NND with hydrophobic propyltrimethoxysilane (PTMS) through co-condensation to decrease the hydrophilicity of the material. A heteropolysiloxane (NND/PTMS) consisting of 70 mol% NND and 30 mol% PTMS was obtained, and the heteropolysiloxane was then utilized as the sensitive material in a 330 MHz SAW sensor capable of detecting SO_2_ in an RH 60% environment. In 2001, Penza et al. [55] developed resonator SAW sensors and surface transverse wave sensors with operating frequencies of 433.92 MHz and 380.0 MHz, respectively; these sensors utilized “rod-like” polymers, as shown in Figure 8, such as poly(bis(tributylphosphine)-platinum-diethynylbiphenyl) (Pt-DEBP), poly-2,5-dibutoxyethynylbenzene (DBEB), and poly-2,5-dioctyloxyethynylbenzene (DOEB), as sensitive materials. The SAW sensors generally outperformed the surface transverse wave sensors, particularly the SAW sensors with Pt-DEBP, which achieved lower detection limits of 2 ppm for SO_2_ and 1 ppm for H_2_S.

In 2005, Jakubik et al. [56] designed a dual delay line sensor with polyaniline as the sensitive film to detect acidic gases. However, they did not obtain a satisfactory response in detecting SO_2_ and H_2_S; the main reason was the thickness of the polyaniline film, which was 100 nm and was inadequate for adsorbing SO_2_ and H_2_S. In 2009, Wen et al. [13] utilized polyaniline as a sensitive material and opted for a film thickness of 120 μm to design a SAW dual delay line sensor consisting of three IDTs and two multistrip couplers. This design not only mitigated the impact of the environment, but also suppressed the generation of bulk acoustic waves (BAW), which ensured precise detection. The sensor also exhibited excellent linearity and sensitivity of 6.8 kHz/ppm over a measurement range from 312 ppb to 20 ppm. Reliable repeatability and long-term stability during testing SO_2_ were displayed. In the same year, Wen et al. [57] developed a dual delay line SAW sensor by utilizing carbon nanotube polyaniline as the sensitive material, based on their previous research. Compared to the pure polyaniline sensor, the carbon nanotube polyaniline sensor exhibited superior linearity, better sensitivity, and a lower detection limit at low concentrations. A sensitivity of 8.3 kHz/ppm and a detection limit of 0.12 ppb were obtained in the concentration range of 31.2 ppb to 20 ppm. The study determined that the application of polyaniline-coated carbon nanotubes solved the problem of pure carbon nanotubes, which tended to aggregate, so the specific surface area of polyaniline was enhanced.

**Figure 9:**
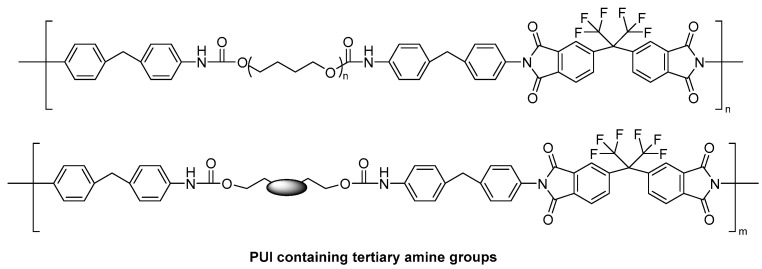
Synthesis of PUIs [58]. Reproduced with permission from Ismaïl Ben Youssef, Functional poly(urethane-imide)s containing Lewis bases for SO_2_ detection by Love surface acoustic wave gas micro-sensors; published by Sensors and Actuators B: Chemical, 2013.

In 2013, Ben et al. [58] created new polyurethane imides (PUIs) with Lewis base properties by synthesizing them with N-methyldiethanolamine (MDEA), N-tert-butyldiethanolamine (tBu-DEA), N-phenyldiethanolamine (Ph-DEA), and 1, 4-diethanolpiperazine-diol (Piperazine-diol) as functional monomers. Figure 9 illustrates the polymer structure. SO_2_ gas was detected accurately at a concentration of 28 ppm by a three-layer Love wave sensor, the sensitivity of sensors utilizing various functional monomers could be enhanced using the following sequence: Piperazine-diol < tBu-DEA ≈ Ph-DEA << MDEA. It was found that the influence of steric hindrance on the sensitivity of the sensor was much higher than that of the alkalinity of the amino group in the functional monomer. Up to now, there have been few reports on the detection of SO_2_ by SAW sensors. In addition to the polymers discussed above, many other sensitive materials have also been used to detect SO_2_, such as metal oxides [59], metal sulfides [60], and small organic molecules [61,62].

### 3.2. Sensitive Functional Materials for H_2_S Detection

**Figure 10:**
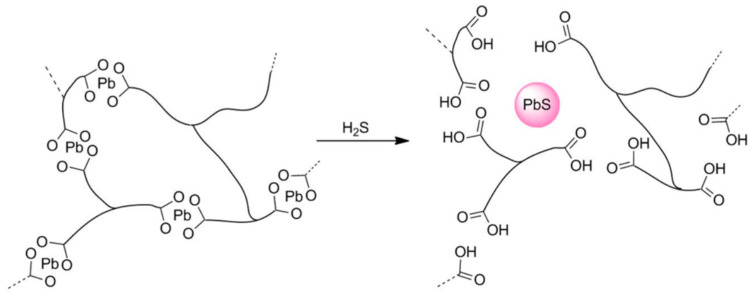
Response mechanism of network polymer [63]. Reproduced with permission from David Rabus, Subsurface H_2_S detection by a surface acoustic wave passive wireless sensor interrogated with a ground penetrating radar; published by ACS Sensors, 2020.

In 2001, Penza et al. [55] used a “rod-like” polymer poly(bis(tributylphosphine)-platinum-diethynylbiphenyl) (Pt-DEBP) as a sensitive material to detect H_2_S, and a detection limit of 1 ppm was obtained. In 2005, Jakubik et al. [56] demonstrated that sensors utilizing polyaniline films had a suboptimal response to H_2_S gas during the testing of acidic gases. The sulfur atom in the H_2_S molecule exhibits distinct reactivity towards metal ions, such as Pb^2+^ or Zn^2+^, based on this particular property. In 2020, Rabus et al. [63] synthesized a network polymer that incorporated Pb^2+^, and used it as sensitive material in a system which could detect H_2_S underground. The recognition capability was enhanced by the specific amalgamation of the lead ion with H_2_S, as shown in Figure 10. Because of the irreversible reaction, its application was limited. In addition to polymers, many other sensitive films, such as metal oxides [64,65], small organic molecules [66,67], carbon nanotubes [18], and ionic liquids [68,69] have also been reported.

## 4. Conclusions

This paper carried out a systematic discussion of polymer materials used in SAW sensors for detecting sulfur-containing toxic or harmful gases. The polymers discussed in this paper can be categorized into carbon-chain polymers and hetero-chain polymers based on their main chain structure, which could be modified by the insertion of functional monomers or functional groups. The sensitive materials are summarized in Table 3. Great progress has been made in the research on polymers for detecting sulfur-containing gases, and there have been many reports on the structure design, selectivity, stability and anti-interference ability of polymers, but determining how to obtain polymer materials with more selectivity for target gases is still the focus of current research. In some cases, due to the similar chemical structure of the measured gas, it is very difficult to accurately identify the target gas with a single polymer material; to solve this problem, SAW sensor arrays and pattern-recognition algorithms are always used to improve the accuracy of detection. In addition, environmental factors including temperature, humidity, and interference gases might affect the sensor during gas detection; therefore, determining how to improve the environmental adaptability of polymer materials to obtain new polymer materials is still a focal area of research.

## Figures and Tables

**Table 1:** Chemical warfare simulants.

Simulant	Simulated Chemical Warfare Agent (CWA)	Median Lethal Dose (LD_50_) Inhaled (ppm)	Ref.
Dimethyl methylphosphonate (DMMP)	Sarin (GB)	18	[25]
Dipropylene glycol monomethyl ether (DPGME)	Nitrogen mustard (HN)	180	[25]
Chloroethyl ethyl sulfide (CEES)	Distilled mustard (HD)	140	[26]
Dibutyl sulfide (DBS)	Distilled mustard (HD)	140	[27]
Chloroethyl phenyl sulfide (CEPS)	Distilled mustard (HD)	140	[27]
1,5-Dichloropentane (DCP)	Distilled mustard (HD)	140	[25]
Dimethylacetamide (DMA)	Distilled mustard (HD)	140	[25]
1,2-Dichloroethane (DCE)	Distilled mustard (HD)	140	[25]
	Soman (GD)	6	[25]
Dichloromethane (DCM)	Phosgene (CG)	800	[25]

**Table 2:** LESR regression coefficients for PECH.

Polymer	Abbr.	Method	*c*	*r*	*s*	*a*	*b*	*l*	*R*	Std Error
Poly(epichlorohydrin)	PECH	SAW	−0.75	0.44	1.44	1.49	1.3	0.55	0.993	0.11

**Table 3:** Summary of polymers used for detecting sulfur-containing compounds with SAW gas sensors.

Device	Polymer Types	Coating Method	Analytes	Sensitivity or Limitation of Detection	Range of Detection	Advantages	Disadvantages	Ref.
158 MHz four-channel SAW delay line sensor array	1. PECH; 2. PEI; 3. ECEL; 4.FPOL.	Spray -coating	HD	0.5 mg/m^3^ in 2 min	2 mg/m^3^ to 50 mg/m^3^	Pattern-recognition algorithms correctly classified the analytes	Influenced by humidity	[32]
159 MHz SAW dual delay line	PECH	Solvent evaporation	HD	48.26 Hz·L·μg^−1^ and 2 mg/m^3^	10 mg/m^3^ to 200 mg/m^3^	Response to HD was 5.6 times greater than that to GB	Not mentioned	[33]
159 MHz SAW dual delay line	PECH	Solvent evaporation	HD	170.1 Hz·m^3^/mg and 2 mg/m^3^	2 mg/m^3^ to 200 mg/m^3^	Good thermal stability, reproducibility, and linear range.	Not mentioned	[34]
159 MHz SAW dual delay line	PECH	Solvent evaporation	CEES	1.62 Hz·L·μg^−1^	5 mg/m^3^ to 100 mg/m^3^	CEES was detected at low concentration.	Influenced by temperature	[35]
200 MHz four two-port SAW resonator array	1. PECH; 2. SE-30; 3. BSP3; 4. PTFP.	Spin-coating method	HD	Not mentioned	Not mentioned	Combined with PNN, the analytes were classified	Not mentioned	[37]
200 MHz two-port SAW resonator	PECH	Spin-coating method	HD	106 Hz/(mg/m^3^) and 0.3 mg/m^3^	1.2 mg/m^3^ to 61.6 mg/m^3^	Good reversibility, stability, reproducibility, and anti-interference ability	Not mentioned	[14]
157 MHz six-channel SAW delay line sensor array	1. PECH; 2. PCPMS 3. Carbowax; 4. PDMS; 5. PEI; 6. PMFTPMS	Not mentioned	DMA DCE	Not mentioned	100 ppm to 250 ppm (DCE)	Combined with PCA, the simulants were well classified	Not mentioned	[38]
157 MHz six-channel SAW delay line sensor array	1. PECH; 2. PCPMS; 3. Carbowax; 4. PDMS; 5. PEI; 6. PMFTPMS.	Spray -coating method	DMA DCP DCE	Not mentioned	30 ppm to 150 ppm (DMA); 80 ppm to 250 ppm (DCE); 5 ppm to 100 ppm (DCP).	The array showed very good sensitivity and specificity rates	DCE and DCM cannot be classified	[25]
Six-channel Love wave delay line sensor array	1. PECH; 2. PCPMS; 3. Carbowax; 4. PDMS; 5. PEI; 6. PMFTPMS.	Spin-coating method	DMA DCE DCP	25 ppm (DMA); 75 ppm (DCE); 5 ppm (DCP).	25 ppm to 250 ppm (DMA); 75 ppm to 250 ppm (DCE); 5 ppm to 25 ppm (DCP).	Good sensitivity and discrimination	Guiding layer will result in damping	[39]
Six-channel Love wave delay line sensor array	1. PECH; 2. PCPMS; 3. Carbowax; 4. PDMS; 5. PEI; 6. PMFTPMS.	Spray -coating method	DMA DCE DCP	15 ppm (DMA); 75 ppm (DCE); 5 ppm (DCP).	15 ppm to 200 ppm (DMA); 75 ppm to 300 ppm (DCE); 5 ppm to 25 ppm (DCP)	Good linearity, stability, reversibility, and accuracy; fast response; high sensitivity and selectivity	Not mentioned	[40]
300 MHz SAW dual delay line	PECH	Solvent evaporation	HD	25 Hz/(mg/m^3^) and less than 2 mg/m^3^	2 mg/m^3^ to 200 mg/m^3^	New phase-modulation methods and design resulted a great improvement in frequency stability	Not mentioned	[41]
3D nanocluster resonator sensors modified by ZnO	1.PECH; 2.SE-30; 3. PTFP; 4. BSP3.	Not mentioned	HD	Not mentioned	Not mentioned	High targeting capacity and disturbance resistance	Greater insertion loss	[42]
300 MHz five- channel two-port SAW resonator array	1. TEA; 2. PECH; 3. SXFA; 4. L-glutamic acid hydrochloride	Dipping method	CEES	14.9 Hz/ppm and less than 0.59 ppm	0.59 ppm to 14 ppm	Combined with pattern-recognition algorithms, analytes were detected within a range of 300 m	Not mentioned	[43]
150 MHz SAW dual delay line	PECH	Not mentioned	CEES	233.17 Hz/(mg/m^3^) and 1.5 mg/m^3^	1.2 mg/m^3^ to 10 mg/m^3^	High response at high humidity	Influenced by temperature	[6]
200 MHz SAW delay line	PECH	Spin-coating method	CEES	1.13 mV/(mg/m^3^) and 0.85 mg/m^3^	1.9 mg/m^3^ to 19.6 mg/m^3^	High sensitivity	Sensor poisoning at high concentration	[44]
SAW dual delay line	PdPc_0.3_PANI_0.7_	Vacuum-coating method	HD	105 kHz/(mg/m^3^)	1.5 mg/m^3^ to 7.5 mg/m^3^	The principle and method were feasible	The mechanism is unknown	[11]
The 163 MHz six-channel SAW delay line sensor array	1. PVA; 2. PVP; 3. PS; 4. PVA+SnCl_4_; 5. PVA+SnCl_4_ 4-h 450 °C; 6. PS+PSMA	Electrospinning technology	DMA; DCE.	Not mentioned	50 ppm to 200 ppm (DCA) 100 ppm to 500 ppm (DCE)	The array achieved a resolution probability of 100% by PCA	Not mentioned	[46]
434-MHz two-port SAW resonator	PLF	Spray-coating	HD CEES DCP	0.01 mg/m^3^ and 2.842 kHz/(mg/m^3^) (CEES);	1 mg/m^3^ to 20 mg/m^3^ (CEES)	Significant response to CEES	Minimal response to HD and DCP	[47]
330 MHz SAW sensor	NND/PTMS	Not mentioned	SO_2_	Not mentioned	Not mentioned	The co-condensation of NND with PTMS reduced the humidity affinity	Not mentioned	[54]
433.92 MHz SAW resonator	1. Pt- DEBP; 2. DBEB; 3. DOEB	Spin-coating method	SO_2_; H_2_S.	2 ppm for SO_2_ and 1 ppm for H_2_S (Pt-DEBP)	1 ppm to 10 ppm	High sensitivity	Not mentioned	[55]
101.764 MHz SAW dual delay line	Polyaniline	Not mentioned	SO_2_	6.8 kHz/ppm	312 ppb to 20 ppm	New design eliminated the external perturbations and suppressed the BAW	Not mentioned	[13]
The SAW dual delay line	Carbon nanotube polyaniline	Solvent evaporation	SO_2_	0.12 ppb and 8.3 kHz/ppm	31.2 ppb to 20 ppm	Superior linearity, better sensitivity, and lower detection limit at low concentration of SO_2_	Not mentioned	[57]
Love SAW microsensor	PUIs (MDEA, tBu-DEA, Ph-DEA, and Piperazine-diol)	Spin-coating method	SO_2_	Not mentioned	Not mentioned	The sensitivity could be changed by changing the amino steric hindrance	The response is not completely reversible	[58]
380 MHz SAW resonator sensor	a network polymer that incorporated Pb^2+^	Spin-coating method	H_2_S	Not mentioned	Not mentioned	High response at high humidity	Response is irreversible	[63]

## Data Availability

Data are contained within the article.
